# Investigating the effect of the inhibitory peptide on *L.monocytogenes* cell invasion: an in silico and in vitro study

**DOI:** 10.1186/s13099-023-00576-7

**Published:** 2023-10-25

**Authors:** Ali Shivaee, Sara Bahonar, Mehdi Goudarzi, Ali Hematian, Bahareh Hajikhani, Behrooz Sadeghi Kalani

**Affiliations:** 1 Department of Medical Microbiology, School of Medicine, Shahid Beheshti University of Medical Sciences, Tehran, Iran; 2 Department of Medical Microbiology, School of Medicine, Ilam University of Medical Sciences, Ilam, Iran; 3 Infectious Diseases and Tropical Medicine Research Center, Shahid Beheshti University of Medical Sciences, Tehran, Iran

**Keywords:** *L.monocytogenes monocytogenes*, Internalin A, E-cadherin, Molecular docking, Inhibitory peptide, Cell line

## Abstract

**Aims:**

*L.monocytogenes monocytogenes* is an omnipresent bacterium that causes a fatal food-borne illness, listeriosis. The connection of this bacterium to E-cadherin through internalin A plays a significant role in the internalization of the bacteria. In this study, this interaction has been investigated for the design of an inhibitory peptide.

**Methods:**

The interaction of the proteins involved in the entry of bacteria was evaluated by molecular docking. According to their interactions, an inhibitory peptide was designed to bind to internalin A by server peptiderive. Its effects on *L.monocytogenes* invasion on the Caco-2 cell line and biofilm formation were also assessed.

**Findings:**

Docking results showed that the peptide has a high affinity for binding to Internalin A. The synthesized peptide at a concentration of 64 µg/ml inhibited 80% of the invasion of *L.monocytogenes* into the Caco-2 cell line. Furthermore, the studied peptide at the highest concentration had a slight inhibitory effect on biofilm formation.

**Conclusion:**

These results reveal that short polypeptides can impede the invasion of target cells by *L. monocytogenes* in vitro and could be advantageous as restoring agents in vivo.

## Introduction

*Listeria monocytogenes* bacteria is a deadly agent transmitted through food, which has caused many concerns in the food industry and medical centres [1, 2]. Its preponderance in food processing facilities is attributed to its endurance to environmental tension, proficiency to reproduce at low temperatures, and capability to form biofilms on different places [3]. This rod-shaped bacterium is an opportunistic intracellular pathogen and one of the critical causes of food-borne human illnesses worldwide [1, 2]. This bacterium is the cause of listeriosis in pregnant women, people with immune system defects, babies and old people. The main transmission route of the bacteria is through consuming contaminated food.

Despite the sensitivity to common antibiotics, treating listeriosis in susceptible people is challenging because it quickly penetrates and spreads to the underlying tissues and escapes the reach of antibiotics and the immune system’s response [3–5].

Initiation of infection depends on the capacity of *L.monocytogenes* to yield its internalization into non-phagocytic cells. The interaction of the *L.monocytogenes* surface protein, internalin InlA mainly mediates this method of entry with its human cell-cell adhesion receptor, E-cadherin. Animal models study shows that the binding of InlA to E-cadherin increases intestinal barrier crossing. Research with cultured human cells has shown that InlA-mediated *L.monocytogenes* entry involves bacterial exploitation of the host’s actin cytoskeleton [3, 5–7]. The interaction of InlA with E-cadherin causes actin induction at the zones of bacterial connection to the host cell membrane [3, 5–7]. Filament assemblage is assumed to aid entry by generating a bulging force that moves the plasma membrane near adhesive bacteria—two ways authority actin polymerization during InlA-mediated *L.monocytogenes* uptake. One pathway is mediated by the host Arp2/3 complex and its activators, including the GTPase, Rac1, and the nuclear-stimulating factor, corectin. The second pathway involves human factors that generally control endocytosis, including the coat protein, clathrin, and the GTPase, dynamin. A crucial unresolved query is whether host procedures separate from actin polymerization are selected by *L.monocytogenes* to boost InlA-mediated entry [4, 6, 7]. One such process could be polarized exocytosis, the localized transport of intracellular vesicles to specific locations on the plasma membrane. Membrane flux through exocytosis can influence the entry of *L.monocytogenes* by helping to regenerate the host cell surface [3–7], whether InlA-mediated entry of *L.monocytogenes* applies the exocyst to promote exocytic membrane trafficking and subsequent plasma membrane remodelling. The main path of the pathogenesis of this bacterium is through internalin A so that mutants lacking this gene or defective in it cannot cause a successful disease [4–7]. Therefore, in this study, according to the importance of internalin A, its interaction with the extracellular domain (ECD) of E-cadherin was investigated, an interfering peptide was designed and manufactured, and its effect on *L.monocytogenes* invasion was evaluated in cell culture. It has been taken that it can be used as a novel alternative method in controlling this bacterium in the future.

## Materials and methods

### The studied strain

In this study, the standard strain of *L. monocytogenes* ATCC19115 was used.

### Extraction of the structure file of internalin A and E-cadherin proteins

For this purpose, the 3D structure of these two proteins is extracted from the Protein data bank database ( RCSB ) [8] and evaluated with Pymol software [9]. The ligand and its additional compounds are removed.

### Investigating the interaction of internalin A and E-cadherin with molecular docking

The HADDOCK 2.4 server [10] was used for molecular docking, and the interaction between the above two proteins was checked with visualization software such as Ligplot [11]. The residues involved in the interaction and their binding energy were evaluated.

### Design of inhibitory peptide

Short-inhibitory peptides were predicted and modified by the Peptiderive server [12] based on the residues involved in the interaction of internalin A and E-cadherin receptors, and the designed peptides were modelled and docked with the E-cadherin receptor. Finally, the peptide with the highest affinity to internalin A was selected and synthesized for in-vitro examination. The peptide construct was synthesized by solid-phase synthesis (GenScript, New Jersey, United States). Also, The physicochemical characteristics of the designed peptide were analyzed using the ProtParam program provided by Expasy.

### Investigating the effect of the inhibitory peptide on bacterial survival

Minimum Inhibitory Concentration (MIC) was used to determine possible lethal effects on *L. monocytogenes* bacteria. The antimicrobial activity of the desired peptide was tested against the *L. monocytogenes* culture by the micro-broth dilution method using 96 U-shaped wells microdilution plates [13]. Accordingly, after adding 100 µL of Trypticase Soy Yeast Extract (TSYE) broth (Oxoid, United Kingdom) to each well of a 96-well plate, 100 µL of different concentrations of the studied peptide (5 µg/mL to 640 µg/mL) was added to the first well and serial dilution was prepared. Finally, 100 µL of the overnight culture containing ∼105 cells was added to each well. Plates were incubated at 37 °C for 24 h, and the MICs were determined by direct observation. Each assay was performed in triplicates and repeated at least twice.

### Cell culture invasion assay

To investigate the inhibitory effects of the designed peptide on *L.monocytogenes* cell invasion, the Caco-2 cell line of human epithelial origin purchased from the National Center and Genetic Reserves of Iran was used.

Hence, the cells were harvested from confluent cell cultures and suspended at a concentration of 1 × 10^5^ cells/mL in Dulbecco’s modified Eagle’s medium (DMEM) containing 10% fetal bovine serum and 1% non-essential amino acids. A 24-well tissue culture plate was seeded with 1 mL per well to confluence for 48 h at a final density of approximately 3.5 × 10^5^ cells per well. Immediately concentrations of 8, 16, 32 and 64 µg of the studied peptide were added to the wells. The invasion assays were performed by incubating *L. monocytogenes* with Caco-2 epithelial cells at a ratio of 100:1. The viable count was determined retrospectively by culturing tenfold serial dilution in PBS onto freshly prepared brain heart infusion (BHI) agar plates. *L. monocytogenes* and epithelial cells were co-incubated for 1 h at 37 °C under 5% CO_2_ air atmosphere. To determine the number of the bacterium that had been internalized into epithelial cells, 90 min incubation in DMEM medium containing 150 µg/mL gentamicin (Sigma, St. Louis, Missouri, USA) was performed in each well to kill extracellular bacterial cells. After washing three times with PBS, epithelial cells were then lysed by Triton X-100 (0.5%) to release the intracellular bacterial cells. The number of *L. monocytogenes* that had invaded the cells was determined by plating serial dilutions of the suspensions onto BHI agar plates.

The control sample (untreated) was used as a standard to normalize the results (set as 100%) [14, 15].

### Biofilm assay

According to the protocol, *L. monocytogenes* bacteria were cultured in a TSB (Tryptic soy broth) medium containing 1% glucose to check biofilm formation. Then 200 µl of the bacterial suspension was poured into each well of a 96 plate and set at 37 °C for 24 h, then, the wells were rinsed twice with PBS (Phosphate-buffered saline*)* and stained with 100 µl of 1% crystal violet for 20 min. Then they were rinsed twice with phosphate-buffered saline. 100 µL of 95% ethanol was added and left at room temperature for 16 min. Crystal violet was disbanded, and a microplate reader calculated cell density by measuring OD at 570 nm. Next, by repeating the above experiment, we added different peptide concentrations to the bacterial biofilm and measured the OD of the biofilm compared to the control [16].

### Statistical analysis

Meaningful results were estimated employing a one-way variance analysis (ANOVA) obeyed by Tukey’s *post hoc* test and Dunnett’s test for multiple comparisons (as demonstrated in figure legends). All data examination and statistical graphing were performed using GraphPad Prism 8.

## Results and discussion

How pathogens interact with the host is essential in identifying its control methods. In fact, over the years of evolution, bacteria have learned how to reach the host. The bacterium *L.monocytogenes* is one of these agents whose pathogenesis stages are well identified [17, 18]. This bacterium enters host cells with a mechanism dependent on the interaction of adhesin and receptor with a phenomenon called the zipper mechanism [17–19]. In the gastrointestinal tract and on the surface of epithelial cells, this phenomenon occurs for the entry of *L.monocytogenes* bacteria with the interaction of internalin A and E-cadherin. Internalin A as a protein has 800 amino acids containing 15 leucine-rich regions (LRR); InlA also has an inter-repeat (IR) area demonstrated rudimentary for binding the LRR repeats domain to E-cadherin. E-cadherin negotiates the construction of adherents conjunctions in a Ca2+-dependent [17–19].

**Fig. 1:**
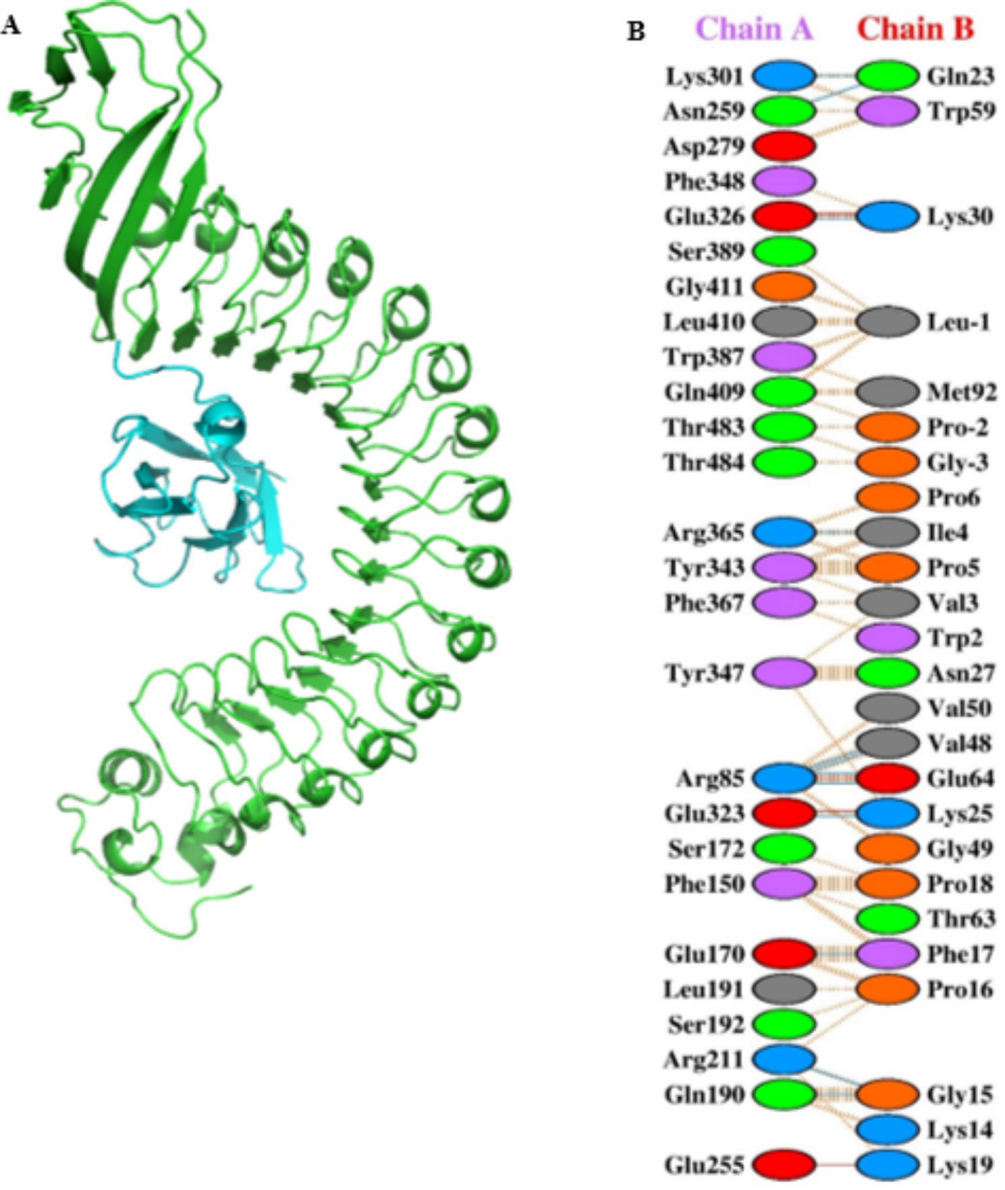
Detailed View of the Interactions between InlA and E-cadherin (-833 Kcal/mol). Cartoon model of the 3D format (**A**) and interactions of InlA with the E-cadherin (**B**). They were interacting residues between InlA and E-cadherin

**Fig. 2:**
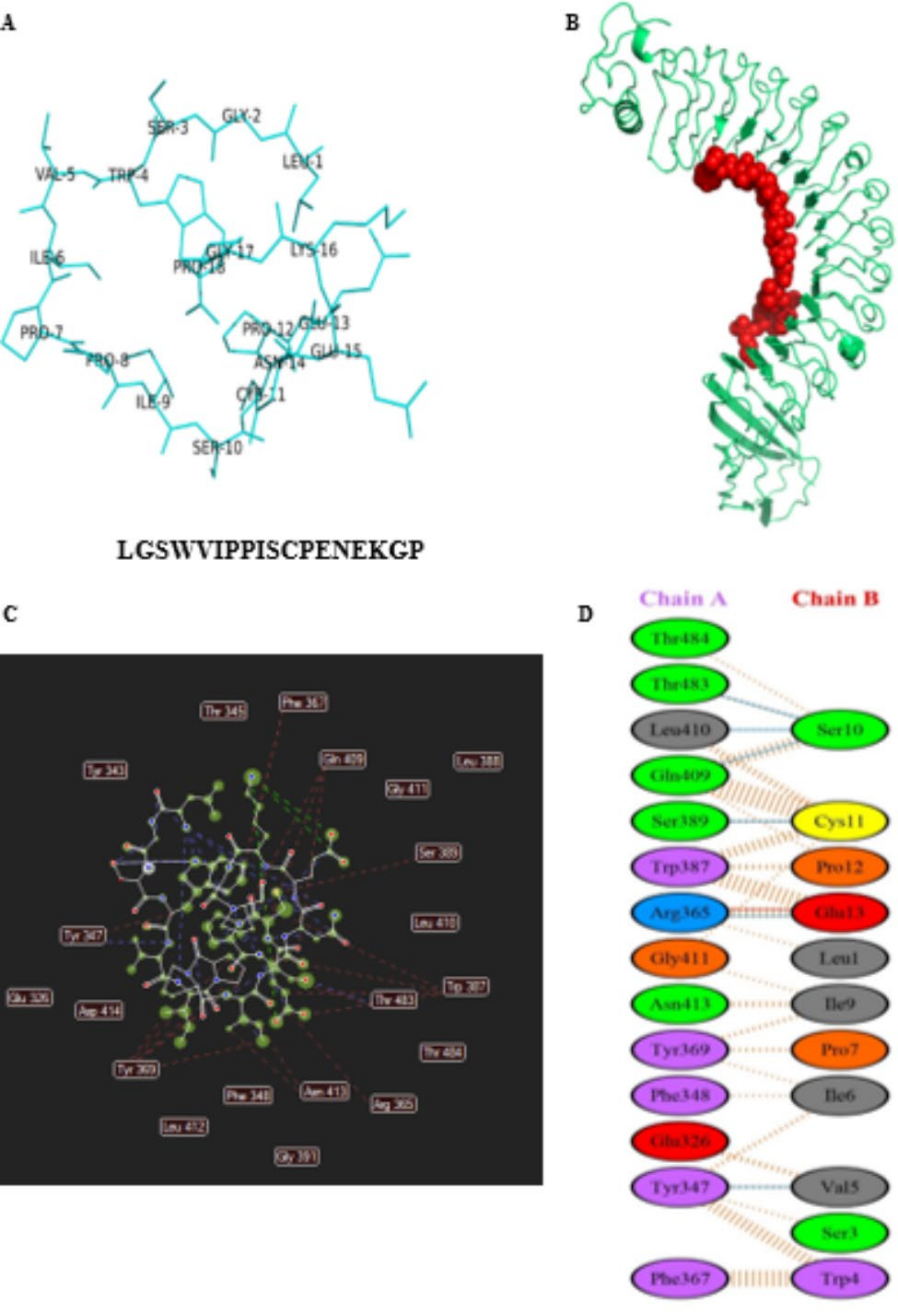
3D structure of studied designed peptide (-851Kcal/mol) (**A**). Cartoon illustration of 3D format and interactions of the designed peptide with the inlA (**B**). They interact residues between the studied peptide and inlA (**C** and **D**)

In this study, this interaction has been used as a target for the design of a drug that may inhibit the entry of bacteria. Figure 1 shows the interaction of internalin A with E-cadherin. Docking results showed that the affinity of this interaction was − 833 Kcal/mol, and many amino acids play a role in it. This complex was introduced to the Peptiderive server, and an inhibitory peptide derived (LGSWVIPPISCPENEKGP) from the interaction of E-cadherin with internalin A was designed. The physicochemical characteristics of the designed peptide has been shown in Table 1. Also, Fig. 2 shows the spatial structure of the studied peptide. Interestingly, the results of docking the peptide in question with internalin A showed that its connection has a greater affinity than the connection of internalin a to E-cadherin (-851 Kcal/mol). The results of this connection and the residues involved in it are shown in Fig. 2.

**Table 1:** The physicochemical characteristics of the studied peptide were analyzed using the ProtParam program provided by Expasy

Property	Measurement
Total Number of Amino Acids	18
Molecular Weight	1923.21
Formula	C87H135N21O26S1
Theoretical pI	4.53
Total Number of Negatively Charged Residues (Asp + Glu)	2
Total Number of Positively Charged Residues (Arg + Lys)	1
Total Number of Atoms	270
Instability Index (II)	47.58
Aliphatic Index (AI)	81.11
Grand Average of Hydropathicity (GRAVY)	-0.256

Determining the minimum inhibitory concentration of the studied peptide revealed that the designed peptide at the highest concentration (640 µg/ml) could not prevent the observable growth of bacteria. Since the studied peptide was not developed against the vital factors for bacterial survival, the results of this part were expected, and the purpose of this part was to the possible effect of the peptide in question on bacterial survival, which could affect the results of cell culture invasion.

**Fig. 3:**
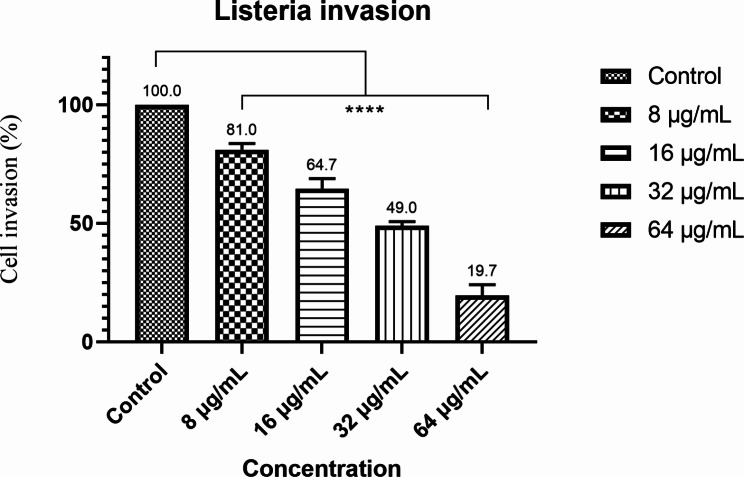
Effect of the designed inhibitory peptide on *L.monocytogenes* invasion. Intracellular *L.monocytogenes* decline after 1 h incubation with 8, 16,32 and 64 µg designed peptide. Untreated bacteria were used as controls. The error bars indicate the SD from three replicate samples. *P ≤ 0.05; **P ≤ 0.01 using one-way ANOVA and Tukey’s test

The results of the inhibitory effect of the studied peptide in inhibiting *L.monocytogenes* in cell culture (Fig. 3) showed that the concentrations of 8, 16, 32, and 64 µg/ml reduced the invasion of *L.monocytogenes* bacteria by 19, 35.3, 51, and 80.3%, respectively, in the Caco-2 cell line (P ≤ 0.0001). The results indicated that the effect of the studied peptide had a direct relationship with the increase in its concentration.

Although the studied peptide at the highest concentration used did not completely inhibit the invasion of bacteria into the cell, the results of this part were promising.

Inhibiting the invasion of *L.monocytogenes* bacteria has been one of the goals of researchers so that, Saura C. Sahu, et al. showed in 2007 a Synthetic polypeptide of 30 aa encompassing Position 16 of human and mouse E-cadherin could inhibit invasion in the human-origin Caco-2 and HepG2 cell lines. They reported that the anti-invasion effect was concentration-dependent, with 100 µg peptide ml − 1 showing a 99% inhibition of invasion, whereas 10 µg ml − 1 resulted in approximately 50% inhibition [20].

Also, in 2019, an interesting study by Moloko G. Mathipa et al. showed that a *Lactobacillus casei* expressing internalin A and B can prevent the invasion and damage of *L.monocytogenes* bacteria to the Caco 2 cell line. The recombinant *L*. *casei* expressing InlAB shows potential for use as a prophylactic intervention strategy for targeted control of *L*. *monocytogenes* during the intestinal phase of infection.

The critical point in this invasion inhibition method is that the probability of developing antimicrobial resistance by bacteria is theoretically significantly lower because bacteria have been able to access the target cells through the evolution of invasion methods over hundreds of years, and the slightest change in the structure of adhesins can disrupt bacterial pathogenesis.

**Fig. 4:**
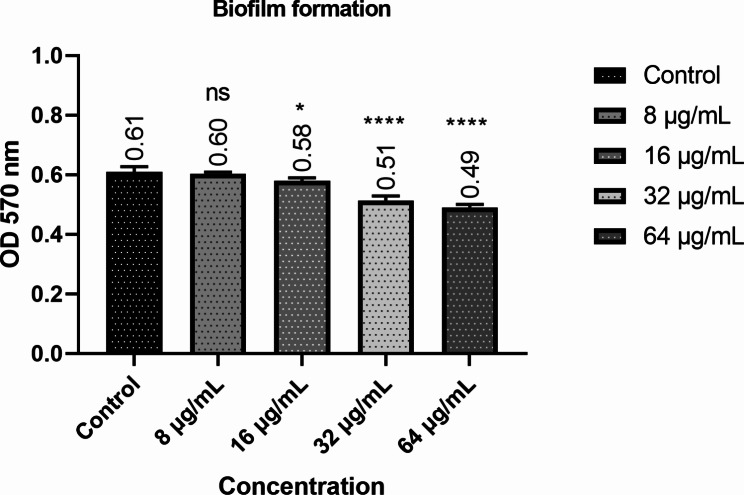
The results of the antibiofilm effects of the designed peptide in different concentrations. Untreated bacteria were used as controls. The error bars indicate the SD from three replicate samples. *P ≤ 0.05;**P ≤ 0.01 using one-way ANOVA and Tukey’s test

In the continuation of the work, the effect of the desired peptide on the biofilm formation of the studied bacteria was investigated. This case was also investigated since internalin A is essential in forming *L. monocytogenes* biofilm. The results of this part revealed that the designed peptide does not have many anti-biofilm properties, and only the concentrations of 32 and 64 µg slightly reduce biofilm formation (Fig. 4).

In the last few decades, the subject of antimicrobial resistance has questioned the extermination of bacterial diseases [21–23]. Antibiotic resistance owing to mutation or the addition of resistance genes may happen nevertheless of antibiotic exposure. Nonetheless, these agents generate a theatrical boost in the emersion of resistant bacteria. This highlights the need to design novel antibacterial agencies [21, 22]. Despite many efforts to improve existing antimicrobials, only a few are effective against resistant bacteria [4]. One applicable strategy to crush this trouble is the designation of conceivable bacterial targets for inventing appropriate medicinal agencies. Protein-protein interactions (PPIs) are crucial in many biological functions and are connected with cancers and communicable illnesses [24]. Consequently, targeting PPIs can be a fortunate therapeutic procedure.

The results of this preliminary study conduct that disease can be averted by preventing the interaction of microbes with host cells, and due to the spread of antimicrobial resistance, this method can be used as an alternative or supplementary method in the future.

Synthetic polypeptides can quickly be constructed and purified in large quantities, so they may help investigate the mechanisms implicated in InlA-mediated host cell invasion.

## Conclusion

These discoveries indicate that small polypeptides can hinder *L.monocytogenes* target cell*s from initiating pathogen*icity and may be worthwhile as restorative agents in vivo, and further studies are thus needed.

## Data Availability

Not applicable.
